# Incidence of SARS-CoV-2 Infection Among People Experiencing Homelessness in Toronto, Canada

**DOI:** 10.1001/jamanetworkopen.2023.2774

**Published:** 2023-03-13

**Authors:** Lucie Richard, Rosane Nisenbaum, Michael Brown, Michael Liu, Cheryl Pedersen, Jesse I. R. Jenkinson, Sharmistha Mishra, Stefan Baral, Karen Colwill, Anne-Claude Gingras, Allison McGeer, Stephen W. Hwang

**Affiliations:** 1 MAP Centre for Urban Health Solutions, Unity Health Toronto, Toronto, Ontario, Canada; 2 Dalla Lana School of Public Health, University of Toronto, Toronto, Ontario, Canada; 3 Harvard Medical School, Boston, Massachusetts; 4 Department of Medicine, University of Toronto, Toronto, Ontario, Canada; 5 Institute of Medical Sciences, University of Toronto, Toronto, Ontario, Canada; 6 Division of Epidemiology and Institute of Health Policy, Management and Evaluation, Dalla Lana School of Public Health, University of Toronto, Toronto, Ontario, Canada; 7 Department of Epidemiology, John Hopkins University, Baltimore, Maryland; 8 Lunenfeld-Tanenbaum Research Institute, Mount Sinai Hospital, Toronto, Ontario, Canada; 9 Department of Microbiology, Mount Sinai Hospital, Toronto, Toronto, Ontario, Canada; 10 Department of General Internal Medicine, University of Toronto, Toronto, Ontario, Canada

## Abstract

**Importance:**

People experiencing homelessness are at high risk of SARS-CoV-2 infection. Incident infection rates have yet to be established in these communities and are needed to inform infection prevention guidance and related interventions.

**Objective:**

To quantify the SARS-CoV-2 incident infection rate among people experiencing homelessness in Toronto, Canada, in 2021 and 2022 and to assess factors associated with incident infection.

**Design, Setting, and Participants:**

This prospective cohort study was conducted among individuals aged 16 years and older who were randomly selected between June and September 2021 from 61 homeless shelters, temporary distancing hotels, and encampments in Toronto, Canada.

**Exposures:**

Self-reported housing characteristics, such as number sharing living space.

**Main Outcomes and Measures:**

Prevalence of prior SARS-CoV-2 infection in summer 2021, defined as self-reported or polymerase chain reaction (PCR)– or serology-confirmed evidence of infection at or before the baseline interview, and SARS-CoV-2 incident infection, defined as self-reported or PCR- or serology-confirmed infection among participants without history of infection at baseline. Factors associated with infection were assessed using modified Poisson regression with generalized estimating equations.

**Results:**

The 736 participants (415 of whom did not have SARS-CoV-2 infection at baseline and were included in the primary analysis) had a mean (SD) age of 46.1 (14.6) years; 486 (66.0%) self-identified as male. Of these, 224 (30.4% [95% CI, 27.4%-34.0%]) had a history of SARS-CoV-2 infection by summer 2021. Of the remaining 415 participants with follow-up, 124 experienced infection within 6 months, representing an incident infection rate of 29.9% (95% CI, 25.7%-34.4%), or 5.8% (95% CI, 4.8%-6.8%) per person-month. Report after onset of the SARS-CoV-2 Omicron variant was associated with incident infection, with an adjusted rate ratio (aRR) of 6.28 (95% CI, 3.94-9.99). Other factors associated with incident infection included recent immigration to Canada (aRR, 2.74 [95% CI, 1.64-4.58]) and alcohol consumption over the past interval (aRR, 1.67 [95% CI, 1.12-2.48]). Self-reported housing characteristics were not significantly associated with incident infection.

**Conclusions and Relevance:**

In this longitudinal study of people experiencing homelessness in Toronto, SARS-CoV-2 incident infection rates were high in 2021 and 2022, particularly once the Omicron variant became dominant in the region. Increased focus on homelessness prevention is needed to more effectively and equitably protect these communities.

## Key Points

**Question:**

What is the period prevalence and incidence of SARS-CoV-2 infection among people experiencing homelessness in Toronto, Canada?

**Findings:**

In this prospective cohort study of 736 people experiencing homelessness in Toronto, 30% of individuals had a history of infection by summer 2021 and a further 30% experienced incident infection within 6 months. Incident infection was significantly associated with reporting after the SARS-CoV-2 Omicron variant became dominant, recent immigration to Canada, and recent alcohol consumption.

**Meaning:**

In this study, people experiencing homelessness in Toronto had high SARS-CoV-2 incident infection rates.

## Teaser

This cohort study examines the SARS-CoV-2 incident infection rate among people experiencing homelessness in Toronto, Canada, in 2021 and 2022 and assesses factors associated with incident infection.

## Introduction

More than 235 000 people experience homelessness in Canada each year.^1^ Homelessness results in reliance on inadequate housing options, many of which are shared, crowded, and/or have high population turnover.^2^ In these settings, where it is difficult or impossible to achieve physical distancing, individuals are at heightened risk for contracting SARS-CoV-2.^2,3,4^ Moreover, people experiencing homelessness have intersecting physical, mental, and social burdens that increase morbidity and mortality relative to housed individuals,^5^ including adverse outcomes following SARS-CoV-2 infection.^5^

To date, seroprevalence estimates from studies describing SARS-CoV-2 infection rates among people experiencing homelessness^3,4,6,7,8,9,10,11,12,13,14,15,16,17,18^ have varied widely, reflecting the timing of data collection, the wider social and policy setting, infection prevention measures in place, and whether outbreaks were under way. These studies similarly show wide variability in individual-, network-, and system-level factors associated with infection, suggesting these too may be context-specific.

Currently, there has been no estimate of the rate of prior SARS-CoV-2 infection among people experiencing homelessness since the emergence of the Omicron variants and no estimates of incident infection (incidence among people without prior history of infection). Thus, in the present study we report, among a random sample of people experiencing homelessness in Toronto, Canada, the period prevalence of SARS-CoV-2 at baseline and incident SARS-CoV-2 infection over 6 months. We also examine characteristics associated with incident infection by 6 months.

## Methods

### Setting and Design

This longitudinal analysis uses data collected between June 2021 and April 2022 from participants of the Ku-gaa-gii pimitizi-win study, a prospective cohort study of people experiencing homelessness in Toronto, a city on Treaty 13 territory in Canada. Ku-gaa-gii pimitizi-win, which translates in English to “life is always/forever moving,” is a spirit name given in ceremony by Elder Dylan Courchene from Anishnawbe Health Toronto to reflect and honor the movement of homeless individuals across the land, the spirit and growth of the land we are on, and the force that connects us all to the future. The Ku-gaa-gii pimitizi-win protocol is available elsewhere.^19^

This study follows the Strengthening the Reporting of Observational Studies in Epidemiology (STROBE) guidelines and received ethics approval from the Research Ethics Board at Unity Health Toronto. All participants provided written informed consent.

Approximately 18 000 individuals experienced homelessness in Toronto in 2021.^20^ A contemporary point-in-time count estimated that 90% of people experiencing homelessness in Toronto are sheltered in emergency and transitional accommodations, with the remainder unsheltered in encampments (informal tent cities) or on the streets.^21^ At the pandemic’s onset, a series of infection prevention strategies were implemented to protect individuals experiencing homelessness in Toronto, including enhanced infection prevention and control funding, routine screening and testing at shelters, opening infection recovery sites with medical supports, and moving thousands of individuals to temporary shelters (ie, physical distancing hotels) to support physical distancing.^22^

At the time of recruitment (June to September 2021) the SARS-CoV-2 Delta variant (B.1.617.2) was rapidly replacing the Alpha variant (B.1.1.7) in infections in Toronto.^23^ Viral activity was very low over the summer, with slow increases in the fall. However, by December 2021 the Omicron variant BA.1 replaced Delta, increasing from less than 1% to greater than 95% of infections over the month.^24^ Omicron variants BA.1, then BA.2, predominated in a large wave of activity from January to March of 2022.^23^

### Recruitment and Follow-up

Individuals were recruited by random number schedule assigned to beds or rooms of 61 participating shelters and physical distancing hotels from June 16 to September 9, 2021; participants were also recruited from 1 urban encampment. To be eligible, individuals had to be experiencing homelessness; not yet be recruited into the study; be aged at least 16 years old; be willing to conduct follow-up interviews; and provide informed consent. Recruited participants were recontacted, through contact information or personal contacts provided at baseline, for follow-up at 3 months (±45 days) between September 15, 2021, and January 10, 2022, and at 6 months (±45 days) between November 17, 2021, and April 13, 2022. Additional information regarding recruitment procedure, follow-up procedure, and sample size calculation details are provided in our published protocol.^19^ After ascertaining history of SARS-CoV-2 at baseline, participants with a history of infection at baseline were excluded from the remainder of this analysis.

### Participant Characteristics

Participants completed a detailed survey at baseline and follow-up intervals, detailing sociodemographic information (age, gender, citizenship status, immigration history, education level), history of known SARS-CoV-2 infection, and activities and behaviors related to COVID-19 (eg, masking, vaccination) or that have been shown in literature to increase risk for infection (eg, alcohol consumption).^25,26^ Participants also provided a recent housing history, which was used to create housing-related exposure variables believed to increase risk for infection, such as number of moves during an interval, average number of people who shared participant living space, and proportion of time spent in various housing types. The survey was reviewed with community partners and piloted with individuals having lived experience of homelessness to confirm face validity and ensure questions did not cause discomfort or harm.

Participants further provided at each interval (1) a saliva sample (swish and gargle method), tested using standard quantitative reverse transcription–polymerase chain reaction (RT-qPCR)^27^ for evidence of current SARS-CoV-2 infection; and (2) a blood sample (plasma tube [BD 365985] or as a dried blood spot [Whatman 903]), to determine by enzyme-linked immunosorbent assay^28^ the presence of past SARS-CoV-2 infection or vaccination-related antibodies (spike protein trimer, spike protein receptor-binding protein [RBD], and nucleocapsid [NP] antigen). In an initial validation, the combined assays had a sensitivity of 91% and specificity close to 100% for plasma or serum.^28^ A full description of variables used in this study is available in eAppendix 1 in Supplement 1.

### Outcomes

Our 2 outcomes of interest were (1) period prevalence of prior SARS-CoV-2 infection at baseline, defined as the number of participants with evidence of current or prior SARS-CoV-2 infection at baseline over the number of participants overall, and (2) incident SARS-CoV-2 infection by 6 months, defined as SARS-CoV-2 infection any time up to and including active infection during the 6-month interview among participants without history of infection at baseline. We ascertained infection through a combination of self-report and biological samples, as people routinely experience infection without their knowledge^7^ and anti–SARS-CoV-2 antibodies may decay over time.^29^ As further detailed in eAppendix 2 in Supplement 1, participants having a self-reported positive PCR test, a positive PCR test administered during the interview, or at least 2 of 3 anti–SARS-CoV-2 antibodies exceeding positivity thresholds in the blood sample were deemed infected. When vaccinated participants without positive PCR tests had serology results without sufficiently elevated NP antigen levels, we deemed them not infected, as COVID-19 vaccines approved in Canada also increase spike and RBD levels and cannot be used as a surrogate infection measure.^28^

### Statistical Analysis

We provided period prevalence of prior SARS-CoV-2 infection at baseline, incident infection rate, and incident infection rate per person-month (defined as number of infections among participants without history of infection at baseline divided by total person-months of observation time) overall and by reporting period (before and after Omicron variants became dominant). When participants seroconverted without a positive PCR test, observation time was stopped at a randomly assigned day between the first and last days of the interval.^30^ Because our outcome is adjudicated using combined biological and self-report data, we did not adjust for test characteristics; as the tests have high sensitivity and specificity, this would only result in a slight underestimate of rates.^31^ Instead, 95% CIs for rates were calculated using the Wilson Score method for proportions.

We also compared participants uninfected at baseline by incident infection status at 6 months. Because housing and behavioral characteristics were assessed at each interval and could thus vary, unadjusted and adjusted rate ratios (uRR and aRR, respectively) with 95% CIs were assessed on interval-level data using univariable and multivariable modified Poisson regression with generalized estimating equations to account for the correlated nature of repeated measures, offset by the log of person-months of observation time.

Factors with significant differences in univariable models or associated with infection in existing literature (eg, age^11,12^; gender^9,14^; alcohol use^25^) were considered for inclusion in the multivariable model. We further considered housing-related exposure variables as well as interviews occurring after Omicron variants became dominant to account for the large wave of infections and outbreaks that occurred in that period.^23^ Correlation coefficients were estimated using variance inflation factor and polychoric correlation, with coefficients greater than 2.5 and 0.4, respectively, flagged for further review prior to modeling. As missingness was nonexistent in the outcome and very low (<1%) across all other factors, we provide complete-case analysis results, as multiple imputation had inconsequential influence on results.

We conducted all analyses using SAS version 7.1 (SAS Institute). Throughout, *P* < .05 (2-sided) was considered significant. We did not adjust for multiple comparisons, following recommended guidelines.^32^

## Results

Of 2643 randomly selected beds, rooms, or tents, individuals were unavailable (n = 1098) or not present (n = 443) in 1553 instances (Figure). A further 12 individuals were unable to consent, and 354 individuals refused to participate. The final sample included 736 participants at baseline, of whom 224 (or 30.4% [95% CI, 27.4%-34.0%]) had a history of SARS-CoV-2 infection. eAppendix 3A in Supplement 1 compares characteristics by baseline infection history: refugees, individuals with temporary or other legal status, and unvaccinated participants were more likely to be excluded due to baseline history of SARS-CoV-2 infection.

**Figure.**
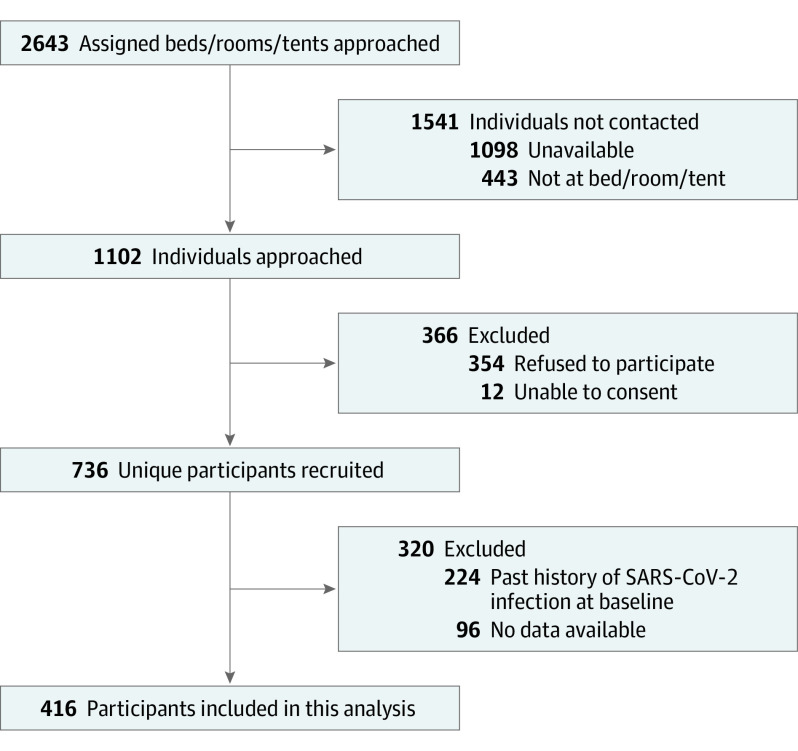
Ku-gaa-gii pimitizi-win Recruitment and Reasons for Nonparticipation or Exclusion From Analysis

Of the remaining 512 participants eligible for this analysis, 96 were lost to follow-up and 1 participant had no useable outcome data. eAppendix 3B in Supplement 1 compares characteristics by loss to follow-up status. Briefly, participants lost to follow-up were younger, less adherent to public health measures, and less likely to be residing in physical distancing hotels. Thus, the final cohort to ascertain incident infection included 415 participants representing a total of 721 intervals and 2136.4 person-months (mean [SD], 5.2 [1.7] months) of observation time.

Characteristics of participants are presented in eAppendix 4 in Supplement 1. Participants had a mean (SD) age of 46.6 (14.5) years. Most participants identified as men (272 of 415 [65.5%]); were Canadian citizens (319 [76.9%]); and were born in Canada (246 [59.3%]). More than 80% of participants reported always or often following each public health recommendation. Overall, participants spent a mean (SD) 57.3% (46.8) of interval days in low-exposures settings, 16.1% (34.3) in moderate-exposure settings, and 25.7% (40.9) in high-exposure settings.

Incident infection over the period and by interval are presented in Table 1. A total of 124 participants became infected by 6 months, representing an overall incident infection rate of 29.9% (95% CI, 25.7%-34.4%) and incident infection rate per person-month of 5.8% (95% CI, 4.8%-6.8%). Approximately 75% of infections occurred in the absence of a positive PCR or rapid antigen test known to the participant, and most (104 [83.9%]) occurred after Omicron variants became dominant. Overall, by spring 2022, at least 47% of the original cohort (348 individuals) had evidence of at least 1 SARS-CoV-2 infection.

**Table 1.** Incident Infection Over Observation Period (0 to 6 months) and by Period, Among Participants Without History of SARS-CoV-2 Infection at Baseline

Follow-up period	No. with outcome information	No. with outcome	No. of outcomes known to participant (% of total outcomes)	% (95% CI)^a^	
				Crude outcome proportion	Outcome rate per person-month
Full observation: baseline (Jun to Sep 2021) to 6 mo (Nov 2021 to Apr 2022)	415	124	32 (25.8)	29.9 (25.7-34.4)	5.8 (4.8-6.8)
Follow-up					
Before SARS-CoV-2 Omicron variant^b^	410	20	2 (10.0)	4.9 (3.2-7.4)	1.7 (1.1-2.6)
After SARS-CoV-2 Omicron variant^c^	311	104	30 (28.8)	33.4 (28.4-38.9)	11.0 (9.0-13.0)

^a^ The 95% CIs were calculated using the Wilson Score method for proportions.

^b^ Interview occurred before December 31, 2021.

^c^ Interview occurred after or on December 31, 2021.

Table 2 shows the results of unadjusted Poisson models assessing factors potentially associated with incident infection by 6 months. Only a few characteristics were significantly associated with infection. These include response period, with interviewees interviewed after Omicron became dominant being more than 6 times more likely to be infected (unadjusted rate ratio [uRR], 6.46 [95% CI 4.04-10.31]); immigration status, with recent immigrants more likely to become infected than those born in Canada (uRR, 2.12 [95% CI, 1.36-3.31]); consumption of alcohol during the interval, with those reporting consumption having higher rates of incident infection (uRR, 1.58 [95% CI, 1.11-2.26]); and proportion of interval spent in noncongregate homeless shelters (uRR for every 10% increase, 0.93 [95% CI, 0.87-0.99]).

**Table 2.** Unadjusted Modified Poisson Regression With Generalized Estimating Equations Assessing Each Factor Potentially Associated With SARS-CoV-2 Incident Infection by 6 Months, Based on 721 Intervals Among 415 Participants Without History of Infection at Baseline

Characteristic	Unadjusted RR (95% CI)^a^	*P* value^b^
**Participant characteristics at baseline**		
Age	1.003 (0.99-1.02)	.62
Age category, y		
30-49	1 [Reference]	.22
16-29	0.68 (0.38-1.23)	
50-69	0.74 (0.51-1.07)	
≥70	1.24 (0.65-2.36)	
Self-reported gender		
Male	1 [Reference]	.31
Female	0.76 (0.52-1.10)	
Other	0.78 (0.21-2.93)	
Citizenship status		
Citizen	1 [Reference]	.64
Landed immigrant	1.40 (0.87-2.26)	
Refugee claimant	1.05 (0.53-2.10)	
Temporary/other	1.24 (0.45-3.38)	
Immigration history		
Born in Canada	1 [Reference]	.01
Immigrated to Canada >10 y ago	1.47 (1.01-2.15)	
Immigrated to Canada ≤10 y ago	2.13 (1.36-3.31)	
Level of education completed		
Any postsecondary	1 [Reference]	.51
Less than high school	1.26 (0.82-1.93)	
High school	1.21 (0.80-1.81)	
COVID-19 vaccines received before baseline		
Complete primary series (2 dose or 1 dose Johnson & Johnson)	1 [Reference]	.31
None	0.77 (0.48-1.23)	
Incomplete primary series	1.12 (0.73-1.73)	
Complete primary series and booster	0.77 (0.09-6.80)	
**Health behaviors and housing during interval**		
Paid or volunteer work		
No	1 [Reference]	.54
Yes	1.12 (0.78-1.61)	
Alcohol consumption		
No	1 [Reference]	.01
Yes	1.58 (1.11-2.26)	
Alcohol consumption frequency		
Never	1 [Reference]	.03
Monthly or less	1.59 (1.03-2.45)	
2-4/mo	1.94 (1.19-3.16)	
2-3/wk	1.86 (1.09-3.19)	
≥4/wsk	0.90 (0.44-1.86)	
Tobacco consumption		
Yes	1 [Reference]	.35
No	1.18 (0.83-1.69)	
Tobacco consumption frequency		
Never	1 [Reference]	.12
Less than daily	0.78 (0.54-1.12)	
Daily	1.38 (0.79-2.42)	
Consumption of illegal or prescription medication for nonmedical reasons		
No	1 [Reference]	.69
Yes	0.92 (0.61-1.39)	
PHG 1: wears face mask in public		
Good (often or always)	1 [Reference]	.39
Poor (never, rarely or occasionally)	1.29 (0.72-2.28)	
PHG 2: distances in public places		
Good (often or always)	1 [Reference]	.60
Poor (never, rarely or occasionally)	1.15 (0.68-1.97)	
PHG 3: avoids crowded places or gatherings		
Good (often or always)	1 [Reference]	.85
Poor (never, rarely or occasionally)	1.05 (0.67-1.64)	
PHG 4: washes hands with soap/sanitizer several times per day		
Good (often or always)	1 [Reference]	.55
Poor (never, rarely or occasionally)	0.82 (0.42-1.58)	
Report after dominance of SARS-CoV-2 Omicron variants		
No	1 [Reference]	<.001
Yes	6.46 (4.04-10.3)	
Proportion of interval spent in		
Congregate shelter (every 10% increase)	1.04 (0.99-1.07)	.22
Noncongregate shelter (every 10% increase)	0.93 (0.87-0.99)	.02
Physical distancing hotel (every 10% increase)	1.01 (0.98-1.05)	.44
Own home (every 10% increase)	0.97 (0.90-1.04)	.35
High-exposure setting (every 10% increase)^c^	1.02 (0.99-1.06)	.25
Moderate-exposure setting (every 10% increase)^d^	0.95 (0.89-1.00)	.05
Low-exposure setting (every 10% increase)^e^	1.01 (0.97-1.04)	.80
Moves during period, No.	1.01 (0.98-1.05)	.41
Average No. of people who shared living space	1.07 (0.99-1.15)	.09

Abbreviations: PHG, public health guideline; RR, rate ratio.

^a^ Unadjusted RR estimated using modified Poisson regression. Models were fitted with generalized estimating equations to account for the correlated nature of interval-level responses.

^b^ *P* values from likelihood ratio test.

^c^ High exposure includes time residing in a congregate homeless shelter, recovery center, nursing home, jail, or immigration detention center.

^d^ Moderate exposure includes time residing in a physical distancing hotel, noncongregate shelter, transitional housing, rooming house, encampment, on the street, rehabilitation center, hospital, or other settings.

^e^ Low exposure includes time residing in own home, supportive housing, private hotel/motel, or staying with friends and family.

Table 3 shows the results of the multivariable assessment of factors associated with incident infection by 6 months. Response after Omicron became dominant remained highly associated with incident infection (aRR, 6.28 [95% CI, 3.94-9.99]), as was immigration within the past 10 years (aRR, 2.74 [95% CI, 1.64-4.58]) and alcohol consumption in the past 3 months (aRR, 1.67 [95% CI, 1.12-2.48]). Housing-related variables were not significantly associated with incident infection.

**Table 3.** Multivariable Modified Poisson Regression With Generalized Estimating Equations Assessing Factors Associated With SARS-CoV-2 Incident Infection by 6 Months, Based on 721 Intervals Among 415 Participants Without History of Infection at Baseline^a^

Characteristic	aRR (95% CI)^b^	*P* value^c^
Age category at index		
30-49 y	1 [Reference]	.13
Younger: 16-29 y	0.59 (0.31-1.14)	
Older: 50-69 y	0.93 (0.62-1.38)	
Older: ≥70 y	1.95 (0.94-4.06)	
Report after dominance of SARS-CoV-2 Omicron variants		
No	1 [Reference]	<.001
Yes	6.28 (3.94-10.0)	
Self-reported gender		
Male	1 [Reference]	.50
Female	0.79 (0.53-1.18)	
Other	0.81 (0.12-5.54)	
Immigration history		
Born in Canada	1 [Reference]	.01
Immigrated to Canada >10 y ago	1.22 (0.81-1.83)	
Immigrated to Canada ≤10 y ago	2.74 (1.64-4.58)	
Alcohol consumption in past interval		
No	1 [Reference]	.01
Yes	1.67 (1.12-2.48)	
Proportion of housing history spent in noncongregate shelter (every 10% increase)	0.96 (0.89-1.03)	.21

Abbreviation: aRR, adjusted rate ratio.

^a^ Listwise deletion: 4 of 721 intervals were excluded due to missing covariate data.

^b^ aRR estimated using multivariable modified Poisson regression. Models were fitted with generalized estimating equations to account for the correlated nature of interval-level responses.

^c^ *P* values from likelihood ratio test.

## Discussion

Nearly one-third of our cohort had prior history of SARS-CoV-2 infection by summer 2021 and an additional 29.9% became infected for the first time during 3- or 6-month follow-up. In total, at least 348 participants out of 736 (or >47% of the cohort) had a history of SARS-CoV-2 infection by spring 2022. In other seroprevalence studies conducted within populations experiencing homelessness, rates range widely, from 4.7% in Denmark to 69.8% in France,^3,4,6,7,8,9,10,11,12,13,14,15,16,17,18^ but these reports are not comparable with ours, as they reflect earlier periods of the pandemic as well as differing local epidemiology and social and policy contexts.

The 2 most applicable, published comparison estimates (both measured using serologic assay data in the broader population) include 1 Canadian report covering the first Omicron wave, which estimated 6-month incident infection at 30% (95% CI, 26%-33%),^33^ and another report from Canadian Blood Services which estimated seroprevalence in Ontario at 4.0% in November 2021, increasing to 34.9% by April 2022.^34^ Additionally, unpublished data from 2 ongoing Toronto-based studies involving education^35^ and health care^36^ workers (which also leveraged combined PCR testing and serology) found that 17.8% and 16.2% of uninfected participants became infected between September 2021 and April 2022 (Brenda L. Coleman, PhD, email, January 19, 2023). If we assume the populations assessed in these reports are not substantially different than Toronto residents generally, we can infer that while incidence rates between people experiencing homelessness and the general population may have become more similar after Omicron variants became dominant,^33^ the overall rate of SARS-CoV-2 incident infection is higher among people experiencing homelessness than among people with stable housing in the region.^33,34,35,36^

The City of Toronto, with prompting by advocates, made significant efforts to implement recommendations^37^ to protect people experiencing homelessness from COVID-19. These interventions included improving infection prevention and control; implementing screening, testing, and vaccination at shelters; opening recovery sites with medical supports; and moving individuals to physical distancing hotels to reduce crowding and support distancing at shelters.^22^ While commendable, these measures focused on mitigation strategies rather than the second series of recommendations targeting upstream factors: namely, decreasing the prevalence of homelessness through appropriately scaled housing strategies. Our results, gleaned from a population benefiting from substantial mitigation efforts, support the conclusion that homelessness may be an independent risk factor for COVID-19 (and future respiratory pandemics), separate from housing-related conditions common to homelessness that are believed to correlate with COVID-19 infection risk.

Our findings also show that incident infection was higher among individuals with a recent history of immigration to Canada. Similar associations were found in population studies, with recent immigrants having higher SARS-CoV-2 infection rates in Canada^38^ and elsewhere.^39^ These studies attribute this finding to the disproportionate representation of immigrants in high-exposure work settings and overcrowded living environments.^38,39^ However, in our study, our closest proxy for overcrowding (average number sharing living space) was not significantly associated with incident infection, and although recent immigrants were more likely to be working in the past interval, work status was not associated with incident infection either. Our findings suggest the reason for the association between recent immigration and risk for infection requires more evaluation: it is possible there is a need to further incorporate modified approaches to mitigation efforts in the shelter and hotel system; it is also possible recent immigration status is correlated with other, unmeasured factors.

Alcohol consumption was also associated with incident infection in our study and has previously been linked with infectious disease susceptibility in the broader population, including for SARS-CoV-2.^25,26^ It has been proposed that because alcohol induces cognitive changes, such changes may lead to exposures that increase risk for SARS-CoV-2 transmission.^25,26^ At the same time, alcohol alters biological susceptibility by impairing immune response.^25,26^ It is unclear from our study whether either or both mechanisms explain this finding or whether alcohol consumption is simply associated with other, unmeasured risk factors.

Finally, we assessed housing-related factors associated with SARS-CoV-2 infection in other studies involving people experiencing homelessness. These factors included average number of contacts per day,^15,18^ average number of people sharing living space,^11^ duration of stay in emergency shelters (both congregate and noncongregate),^9,15,18^ and stay in physical distancing hotels.^40^ Although associations all demonstrated the expected direction (with greater shared or exposed settings having higher rates of incident infection and vice versa), and the proportion of time residing in noncongregate shelters was significantly associated with reduced risk of infection in unadjusted analyses, these were not statistically significant in adjusted analyses. In part, the within-group nature of this analysis (as opposed to directly comparing housing-related factors among people experiencing homelessness as well as those not experiencing homelessness) may have contributed to this result. It is possible exposure summarized over the interval (vs isolating exposure immediately preceding infection events) lessened the effects of these exposures or that our sample was insufficiently powered to identify significant differences, having already excluded 30% of our cohort due to history of infection at baseline. Finally, it is possible that incident infection, as opposed to any occurrence of infection (which would also include reinfections), is unrelated to housing-related exposure because individuals who would have become infected due to housing-related factors already had history of infection by the baseline interview.

### Limitations

This study has limitations. While we randomly recruited from numerous sites across Toronto as well as from one encampment, we were unable to sample from the street or other settings. Approximately 10% of people experiencing homelessness in Toronto are unsheltered^21^; thus, our results approximate, but are not fully representative of, the experience of people experiencing homelessness in Toronto. Furthermore, survey and housing data were self-reported; while reports occurred every 3 months to help prevent issues of increasing unreliability over time, self-report data can suffer from social desirability bias, particularly among populations facing significant stigma.^41^ We also note that the reliance on a single antibody measurement (NP) for evidence of infection in vaccinated individuals may have led to underestimating infections, as sensitivity of NP as a single antigen at the selected cutoff was 79%.^28^

Additionally, while we provide actionable data about SARS-CoV-2 infection among people experiencing homelessness in Toronto, we could not describe reinfections. Reinfection contributes nontrivial additional risks for adverse outcomes, regardless of vaccination^42^; they are also believed to occur more frequently among those experiencing homelessness.^43^ Future work should investigate full infection occurrence rates through combined incident infection and reinfection rates as well as assess the impact of incident infection and reinfection on adverse health outcomes, hospitalization, and mortality.

Furthermore, our recruitment included individuals with a baseline history of infection. While this allowed us to measure period prevalence at baseline, we were obliged to exclude these individuals from the incident infection analysis, which may have created a nonrepresentative incident cohort. A fully random incident cohort might have generated higher rates. A further 97 individuals lost to follow-up were younger, less adherent to public health measures, and spent less time in physical distancing hotels than average (eAppendix 3B in Supplement 1). It is unclear how complete follow-up would have affected incident infection rates or factors associated with incident infection; thus, results should be interpreted with this in mind.

## Conclusions

In this study, people experiencing homelessness in Toronto in 2021 and 2022 had elevated SARS-CoV-2 incident infection rates, potentially reflecting upstream structural risks that make unhoused individuals vulnerable to infection compared with housed counterparts. Among people experiencing homelessness, immigration status and alcohol consumption were associated with higher incident infection by 6 months, suggesting a possible need for modified approaches to infection mitigation efforts in shelters and hotels.

## Notes

Supplement 1. **eAppendix 1.** Full Variable Definitions **eAppendix 2.** Adjudication of Self-report and Serology Results to Determine SARS-CoV-2 Infection **eAppendix 3.** Baseline Characteristics of Participants **eAppendix 4.** Characteristics of Participants Without History of Infection at Baseline, by Incident Infection Status by 6 Months (415 Participants; 716 Intervals)
eReferences.

Supplement 2.
Data Sharing Statement
