# Supplementary material for: Incidence of SARS-CoV-2 Infection Among People Experiencing Homelessness in Toronto, Canada
Source: JAMA Netw Open. 2023 Mar 13;6(3):e232774. doi: 10.1001/jamanetworkopen.2023.2774 (PMC10011938; doi:10.1001/jamanetworkopen.2023.2774)
Supplement: Supplement 1. — eAppendix 1. Full Variable Definitions eAppendix 2. Adjudication of Self-report and Serology Results to Determine SARS-CoV-2 Infection eAppendix 3. Baseline Characteristics of Participants eAppendix 4. Characteristics of Participants Without History of Infection at Baseline, by Incident Infection Status by 6 Months (415 Participants; 716 Intervals) eReferences. [file jamanetwopen-e232774-s001.pdf]

## Supplemental Online Content

Richard L, Nisenbaum R, Brown M, et al. Incidence of SARS-CoV-2 infection among people experiencing homelessness in Toronto, Canada. *JAMA Netw Open*. 2023;6(3):e232774. doi:10.1001/jamanetworkopen.2023.2774

**eAppendix 1.** Full Variable Definitions
**eAppendix 2.** Adjudication of Self-report and Serology Results to Determine SARS-CoV-2 Infection
**eAppendix 3.** Baseline Characteristics of Participants
**eAppendix 4.** Characteristics of Participants Without History of Infection at Baseline, by Incident Infection Status by 6 Months (415 Participants; 716 Intervals)
**eReferences.**

This supplemental material has been provided by the authors to give readers additional information about their work.

© 2023 Richard L et al. *JAMA Network Open.*

## eAppendix 1. Full Variable Definitions

The table below includes all variables included in this analysis (main or supplemental). Included covariates are either shown in existing literature to affect risk for SARS-CoV-2 infection or be related to SARS-CoV-2 related adverse health outcomes (which may also, indirectly, affect behaviours affected risk of infection). In all instances, measures were collected based on a Settler Canadian (Western) understanding of health-related factors and associated prevention guidelines appropriate during the COVID-19 pandemic.

| Variable | Definition |
|---|---|
| **Outcome** | |
| **SARS-CoV-2 period prevalence** | SARS-CoV-2 infection among participants at the baseline interview. Answers include Yes or No. Category is determined using adjudicated participant self-report and biological sample results.[1] |
| **SARS-CoV-2 incident infection** | SARS-CoV-2 infection among participants without a history of infection at the baseline interview any time up to and including active SARS-CoV-2 infection during the 6-month interview. Answers include Yes or No. Category is determined using adjudicated participant self-report and biological sample results.[1] |
| **Covariates** | |
| **Age** | Participant's age as of the date of the baseline survey. Calculated using the date of the survey and the participant's self-reported date of birth. Also expressed as age groups (16 to 29 years; 30 to 49 years; 50 to 69 years; and 70+ years). |
| **Gender** | Participant's self-reported gender, as of the date of the baseline survey. Answers include 'male', 'female', 'other' (which combines 'non-binary', 'gender queer', 'agender', 'transgender' or similar) and 'refused/don't know'. 'Refused/don't know' category is not included in modelling. |
| **Citizenship status** | Participant's self-reported citizenship status, as of the date of the baseline survey. Answers include 'Citizen', 'Landed immigrant' (also known as 'Permanent resident'), 'Refugee', 'Temporary/Other' and 'Refused/Don't know'). 'Refused/don't know' category is not included in modelling. |
| **Immigration history** | Recentness of participant immigration to Canada, as of the date of the baseline survey. Calculated from the Citizenship status and year of immigration variables. Categories include 'Born in Canada (n/a)', '10 or more years ago', and 'Less than 10 years ago'. |
| **Education level** | Participant's self-reported highest level of completed education, as of the baseline interview date. Answers include 'less than high school' (secondary school), 'high school', 'any-post-secondary' (which combines 'vocational/technical school', 'college/university', 'graduate/professional school'), or 'Refused/Don't know'. 'Refused/don't know' category is not included in modelling. |

© 2023 Richard L et al. *JAMA Network Open.*

| Variable | Definition |
|---|---|
| **COVID-19 vaccination** | Participant's receipt of Health-Canada approved COVID-19 vaccine by the baseline interview date. Answers are categorized into either:<br><br>1) One or more doses, or None (unvaccinated); or<br>2) None (unvaccinated), Incomplete primary series (One dose unless the dose is Johnson & Johnson), Complete primary series (2 dose or 1 dose of Johnson & Johnson) or Complete primary series and booster (3+ doses).<br><br>Category is determined using a) participant self-report and b) participant biological sample results.[1] |
| **Hypertension** | Diagnosis by a physician of hypertension at any point prior to the interview, self-reported by the participant. Answers include 'Yes' and 'No'. 'Refused/don't know' category is not included in modelling |
| **Diabetes** | Diagnosis by a physician of diabetes at any point prior to the interview, self-reported by the participant. Answers include 'Yes' and 'No'. 'Refused/don't know' category is not included in modelling |
| **Asthma** | Diagnosis by a physician of asthma at any point prior to the interview, self-reported by the participant. Answers include 'Yes' and 'No'. 'Refused/don't know' category is not included in modelling |
| **Lung disease (COPD, emphysema, or chronic bronchitis)** | Diagnosis by a physician of chronic lung disease (including chronic obstructive pulmonary disease, emphysema or chronic bronchitis) at any point prior to the interview, self-reported by the participant. Answers include 'Yes' and 'No'. 'Refused/don't know' category is not included in modelling |
| **Heart disease (heart attack, heart failure, or coronary artery disease)** | Diagnosis by a physician of heart disease (including heart attack, heart failure or coronary artery disease) at any point prior to the interview, self-reported by the participant. Answers include 'Yes' and 'No'. 'Refused/don't know' category is not included in modelling |
| **History of stroke** | History of stroke at any point prior to the interview, self-reported by the participant. Answers include 'Yes' and 'No'. 'Refused/don't know' category is not included in modelling |
| **Chronic kidney disease** | Diagnosis by a physician of chronic kidney disease at any point prior to the interview, self-reported by the participant. Answers include 'Yes' and 'No'. 'Refused/don't know' category is not included in modelling |
| **Liver disease** | Diagnosis by a physician of liver disease at any point prior to the interview, self-reported by the participant. Answers include 'Yes' and 'No'. 'Refused/don't know' category is not included in modelling |
| **Chronic neurological disorder** | Diagnosis by a physician of chronic neurological disorder at any point prior to the interview, self-reported by the participant. Answers include 'Yes' and 'No'. 'Refused/don't know' category is not included in modelling |
| **Cancer** | Diagnosis by a physician of cancer at any point prior to the interview, self-reported by the participant. Answers include 'Yes' and 'No'. 'Refused/don't know' category is not included in modelling |

© 2023 Richard L et al. *JAMA Network Open.*

| Variable | Definition |
|---|---|
| **HIV/AIDS** | Diagnosis by a physician of HIV/AIDS at any point prior to the interview, self-reported by the participant. Answers include 'Yes' and 'No'. 'Refused/don't know' category is not included in modelling |
| **Immune Suppressed (other than HIV/AIDS)** | Diagnosis by a physician of a non HIV/AIDS-related immune suppression at any point prior to the interview, self-reported by the participant. Answers include 'Yes' and 'No'. 'Refused/don't know' category is not included in modelling |
| **Paid or volunteer work** | Participant's self-reported work experience during the interval. Work includes any form of paid activity, and volunteer work includes any form of unpaid activity on behalf of another person or organization. Answers categorized into 'Yes' and 'No/Refused/Don't know'. 'Refused/don't know' category is not included in modelling. |
| **Alcohol consumption** | Consumption of alcohol by the participant during the interval, irrespective of frequency or quantity of consumption. Categories include 'Yes' and 'No'. 'Refused/don't know' category is not included in modelling. |
| **Frequency of alcohol consumption** | Average frequency of alcohol consumption by the participant during the interval, irrespective of quantity consumed. Categories include 'Never', 'Monthly or less', '2-4 times a month', '2-3 times a week' and '4+ times a week'. 'Refused/don't know' category is not included in modelling. |
| **Tobacco consumption** | Consumption of tobacco by the participant during the interval, irrespective of frequency or quantity of consumption. Categories include 'Yes' and 'No'. 'Refused/don't know' category is not included in modelling. |
| **Frequency of tobacco consumption** | Average frequency of tobacco consumption by the participant during the interval, irrespective of quantity consumed. Categories include 'Never', 'Less than daily', and 'Daily'. 'Refused/don't know' category is not included in modelling. |
| **Consumption of illegal or prescription medication for non-medical reasons** | Consumption of any illegal drug or prescription medication taken for non-medical reasons during the interval. Categories include 'No' or 'Yes'. Refused/don't know category is not included in modelling. |
| **Observance of public health guidelines: wearing a face mask in public places** | The participant's self-reported adherence to public health guidelines active in Ontario during the interval (specifically: the degree to which the participant wears a face mask when in public places). Answers include 'Low' (including Never/rarely/occasionally), 'High' (including 'Often/Always') and 'Refused/Don't know. 'Refused/don't know' category is not included in modelling. |
| **Observance of public health guidelines: distancing in public places** | The participant's self-reported adherence to public health guidelines active in Ontario during the interval (specifically: the degree to which the participant practices physical distancing while in public places). Answers include 'Low' (including Never/rarely/occasionally), 'High' (including 'Often/Always') and 'Refused/Don't know. 'Refused/don't know' category is not included in modelling. |
| **Observance of public health guidelines: avoiding crowded places or gatherings** | The participant's self-reported adherence to public health guidelines active in Ontario during the interval (specifically: the degree to which the participant avoids crowded places or gatherings). Answers include 'Low' (including |

© 2023 Richard L et al. *JAMA Network Open.*

| Variable | Definition |
| --- | --- |
| | Never/rarely/occasionally), 'High' (including 'Often/Always') and 'Refused/Don't know. 'Refused/don't know' category is not included in modelling. |
| **Observance of public health guidelines: washing hands several times per day** | The participant's self-reported adherence to public health guidelines active in Ontario during the interval (specifically: the degree to which the participant washes their hands with soap/hand sanitizer several times per day). Answers include 'Low' (including Never/rarely/occasionally), 'High' (including 'Often/Always') and 'Refused/Don't know. 'Refused/don't know' category is not included in modelling. |
| **Proportion of interval spent in congregate (shared) homeless shelter** | Proportion of the interval spent by the participant in congregate (shared sleeping space) homeless shelter(s). Minimum value = 0%; Maximum value = 100% |
| **Proportion of interval spent in non-congregate (private) homeless shelter** | Proportion of the interval spent by the participant in non-congregate (private sleeping space) homeless shelter(s). Minimum value = 0%; Maximum value = 100% |
| **Proportion of interval spent in physical distancing hotel** | Proportion of the interval spent by the participant in physical distancing hotel(s). Minimum value = 0%; Maximum value = 100% |
| **Proportion of interval spent in own place** | Proportion of the interval spent by the participant in their own home/accommodation (ie. The participant is 'housed'). Minimum value = 0%; Maximum value = 100% |
| **Proportion of interval spent in High Exposure setting** | Proportion of the interval spent by the participant in housing settings considered 'high exposure', ie. At highest risk of contracting SARS-CoV-2 due to crowding, turnover and/or congregate nature of sleeping/living setting(s). Includes the following housing types: congregate homeless shelter, recovery centre, nursing home, jail or immigration detention centre. Minimum value = 0%; Maximum value = 100% |
| **Proportion of interval spent in Moderate Exposure setting** | Proportion of the interval spent by the participant in housing settings considered 'moderate exposure', ie. At less risk than 'High exposure' and greater risk than 'Low exposure' of contracting SARS-CoV-2 due to crowding, turnover and/or congregate nature of sleeping/living setting(s). Includes the following housing types: physical distancing hotel, non-congregate shelter, transitional housing, rooming house, encampment, on the street, rehab, hospital or 'other' settings. Minimum value = 0%; Maximum value = 100% |
| **Proportion of interval spent in Low Exposure setting** | Proportion of the interval spent by the participant in housing settings considered 'low exposure', ie. At least risk of contracting SARS-CoV-2 due to lower crowding, turnover and/or less congregate nature of sleeping/living setting(s). Includes the following housing types: own home, supportive housing, private hotel/motel, or staying with friends and family. Minimum value = 0%; Maximum value = 100% |

© 2023 Richard L et al. *JAMA Network Open.*

| Variable | Definition |
|---|---|
| **Number of moves during the interval** | Number of housing moves by the participant during the interval. Includes changes in residence (new housing episodes) as well as moves indicated by the participant during periods where housing was shared between two or more locations. |
| **Average number of people who shared living space** | Number of people sharing living space with the participants during the interval, on average. Reported in the housing history for each housing episode, this may thus represent the 'average of an average' where number with whom living space is shared fluctuated within a housing episode (for example, in a congregate homeless shelter) as well as between episodes. |
| **Report after onset of the Omicron variant** | Categorization of follow-up interview date as being before December 31 2021 or on or after December 31 2021. The Omicron variant became dominant (>99%) by the end of December, so the end of December was selected to ensure individuals identified as reporting 'during Omicron' were, if infected, almost certainly infected with this variant. |

© 2023 Richard L et al. *JAMA Network Open.*

## eAppendix 2. Adjudication of Self-report and Serology Results to Determine SARS-CoV-2 Infection

Where a positive PCR test was self-reported by the participant, this was accepted as-given. In the absence of a positive PCR test reported by the participant, we relied on detection of SARS-CoV-2 via PCR test administered during the interview [2] or antibodies detected by enzyme-linked immunosorbent assay (ELISA) from plasma and/or dried blood spot samples, also collected during the interview. The methodology regarding the ELISA are more fulsomely described elsewhere.[3] Briefly, the assays detect immunoglobulin (Ig)Gs against the spike protein trimer (SMT1), spike protein receptor-binding protein (RBD) and nucleocapsid protein (NP), with raw values normalised to a blank-subtracted reference point from the reference curve to create relative ratios. These in turn are calibrated to BAU/ml equivalents. Infection was deemed to have occurred where at least two out of three antibodies exceeded the positivity threshold (set at 3 standard deviations from the mean of the log distribution of monitored controls) [3]:

| | **Positivity threshold** |
|---|---|
| Nucleocapsid protein | 34.5 BAU/ml |
| Receptor-binding protein | 31.0 BAU/ml |
| Spike protein | 11.3 BAU/ml |

Because participants may also have received one or more COVID-19 vaccines during the period, which affects RBD and Spike protein levels, we further assessed against self-reported vaccination status when only Spike and RBD were deemed positive; if the individual was vaccinated, we deemed those individuals not infected.

© 2023 Richard L et al. *JAMA Network Open.*

## eAppendix 3. Baseline Characteristics of Participants

### 3A – Baseline characteristics of participants at baseline, by history of infection status (evidence of prior SARS-CoV-2 infection; no evidence of prior infection)

| Participant demographics at baseline | | Total (n=736) | No evidence of past infection (n=512) | Evidence of past infection (n=224) | P-values |
|---|---|---|---|---|---|
| Age, mean (SD) | | 46.08 (14.6) | 45.93 (14.4) | 46.43 (15.1) | 0.788 |
| Age category, N (%) | | | | | |
| | 16-29 years old | 100 (13.59%) | 68 (13.28%) | 32 (14.29%) | 0.634 |
| | 30-49 years old | 326 (44.29%) | 230 (44.92%) | 96 (42.86%) | |
| | 50-69 years old | 263 (35.73%) | 185 (36.13%) | 78 (34.82%) | |
| | 70+ years old | 47 (6.39%) | 29 (5.66%) | 18 (8.04%) | |
| Self-reported gender, N (%) | | | | | |
| | Male | 486 (66.03%) | 334 (65.23%) | 152 (67.86%) | 0.722 |
| | Female | 231 (31.39%) | 163 (31.84%) | 68 (30.36%) | |
| | Other | 17 (2.31%) | 13 (2.54%) | 4 (1.79%) | |
| | Refused/Don't know | 2 (0.27%) | 2 (0.39%) | 0 (0%) | |
| Citizenship status, N (%) | | | | | |
| | Citizen | 564 (76.63%) | 398 (77.73%) | 166 (74.11%) | 0.017 |
| | Landed immigrant | 90 (12.23%) | 66 (12.89%) | 24 (10.71%) | |
| | Refugee claimant | 55 (7.47%) | 33 (6.45%) | 22 (9.82%) | |
| | Temporary/Other | 20 (2.72%) | 11 (2.15%) | 9 (4.02%) | |
| | Refused/Don't know | 7 (0.95%) | 4 (0.78%) | 3 (1.33%) | |
| Immigration history, N (%) | | | | | |
| | > 10 years ago | 187 (25.41%) | 139 (27.15%) | 48 (21.43%) | 0.119 |
| | <= 10 years ago | 108 (14.67%) | 68 (13.28%) | 40 (17.86%) | |
| | N/A (Born in Canada) | 441 (59.92%) | 305 (59.69%) | 136 (60.71%) | |
| Top education completed, N (%) | | | | | |
| | Less than high school | 208 (28.26%) | 149 (29.10%) | 59 (26.34%) | 0.056 |
| | High school | 255 (34.65%) | 183 (35.74%) | 72 (32.14%) | |

© 2023 Richard L et al. *JAMA Network Open.*

| Participant demographics at baseline | Total (n=736) | No evidence of past infection (n=512) | Evidence of past infection (n=224) | P-values |
|---|---|---|---|---|
| Post-secondary | 264 (35.87%) | 173 (33.79%) | 91 (40.63%) | |
| Refused/Don't know | 9 (1.23%) | 7 (1.37%) | 2 (0.89%) | |
| Received one or more COVID-19 vaccine, N (%) | | | | |
| Yes | 585 (79.48%) | 420 (82.03%) | 165 (73.66%) | |
| No | 143 (19.43%) | 85 (16.60%) | 58 (25.89%) | 0.005 |
| Missing serology | 8 (1.09%) | 7 (1.37%) | 1 (0.44%) | |
| **Health behaviors during latest reported interval** | | | | |
| Paid or volunteer work, N (%) | 215 (29.21%) | 148 (28.91%) | 68 (30.36%) | 0.691 |
| Alcohol consumption | 449 (61.01%) | 305 (59.57%) | 143 (63.84%) | 0.269 |
| Alcohol consumption frequency | | | | |
| Never | 282 (38.32%) | 204 (39.84%) | 79 (35.27%) | |
| Monthly or less | 174 (23.64%) | 118 (23.05%) | 55 (24.55%) | |
| 2-4 times a month | 100 (13.59%) | 70 (13.67%) | 30 (13.39%) | 0.688 |
| 2-3 times a week | 79 (10.73%) | 54 (10.55%) | 25 (11.16%) | |
| 4+ times a week | 96 (13.04%) | 63 (12.30%) | 33 (14.73%) | |
| Refused/Don't know | 5 (0.68%) | 3 (0.59%) | 2 (0.89%) | |
| Tobacco consumption | 504 (68.48%) | 355 (69.34%) | 149 (66.52%) | 0.623 |
| Tobacco consumption frequency | | | | |
| Never | 230 (31.25%) | 156 (30.47%) | 74 (33.04%) | |
| Less than daily | 435 (59.10%) | 303 (59.18%) | 132 (58.93%) | 0.601 |
| Daily | 69 (9.38%) | 52 (10.16%) | 17 (7.59%) | |
| Refused/Don't know | 2 (0.28%) | 1 (0.20%) | 1 (0.44%) | |
| PHG #1: wears face mask in public | | | | |
| Good (Often or Always) | 651 (88.45%) | 455 (88.87%) | 196 (87.50%) | |
| Poor (Never, Rarely or Occasionally) | 83 (11.28%) | 56 (10.94%) | 27 (12.05%) | 0.753 |
| Refused/Don't know | 2 (0.27%) | 1 (0.20%) | 1 (0.45%) | |
| PHG #2: distances in public places | | | | |
| Good (Often or Always) | 625 (84.92%) | 437 (85.35%) | 187 (83.48%) | 0.809 |

© 2023 Richard L et al. *JAMA Network Open.*

| Participant demographics at baseline | Total (n=736) | No evidence of past infection (n=512) | Evidence of past infection (n=224) | P-values |
|---|---|---|---|---|
| Poor (Never, Rarely or Occasionally) | 102 (13.86%) | 69 (13.48%) | 34 (15.18%) | |
| Refused/Don't know | 9 (1.22%) | 6 (1.17%) | 3 (1.34%) | |
| PHG #3: avoids crowded places or gatherings | | | | |
| Good (Often or Always) | 583 (79.21%) | 406 (79.30%) | 176 (78.57%) | |
| Poor (Never, Rarely or Occasionally) | 140 (19.02%) | 97 (18.95%) | 44 (19.64%) | 0.975 |
| Refused/Don't know | 13 (1.77%) | 9 (1.76%) | 4 (1.79%) | |
| PHG #4: washes hands with soap/sanitizer several times per day | | | | |
| Good (Often or Always) | 663 (90.08%) | 461 (90.04%) | 202 (90.18%) | |
| Poor (Never, Rarely or Occasionally) | 70 (9.51%) | 49 (9.57%) | 21 (9.38%) | 0.991 |
| Refused/Don't know | 3 (0.41%) | 2 (0.39%) | 1 (0.45%) | |
| **Housing History in latest reported interval** | | | | |
| % in congregate shelter | | | | |
| Mean (SD) | 26.53 (40.6) | 26.29 (40.1) | 27.07 (41.7) | 0.810 |
| Median (IQR) | 0.0 (0, 57) | 0.0 (0, 54) | 0.0 (0, 71) | 0.837 |
| % in non-congregate shelter | | | | |
| Mean (SD) | 13.46 (31.5) | 13.54 (31.7) | 13.69 (31.6) | 0.953 |
| Median (IQR) | 0.0 (0, 0) | 0.0 (0, 0) | 0.0 (0, 0) | 0.744 |
| % in Physical Distancing Hotel | | | | |
| Mean (SD) | 40.83 (46.3) | 41.73 (46.4) | 38.78 (46.1) | 0.427 |
| Median (IQR) | 5.9 (0, 100) | 7.4 (0, 100) | 3.8 (0, 100) | 0.518 |
| % in own place | | | | |
| Mean (SD) | 5.73 (20.2) | 6.38 (20.8) | 3.83 (18.0) | 0.112 |
| Median (IQR) | 0.0 (0, 0) | 0.0 (0, 0) | 0.0 (0, 0) | 0.012 |
| % in High Exposure[1] setting | | | | |
| Mean (SD) | 27.75 (41.2) | 27.39 (40.7) | 28.56 (42.4) | 0.723 |
| Median (IQR) | 0.0 (0, 69) | 0.0 (0, 62) | 0.0 (0, 80) | 0.744 |
| % in Moderate Exposure[2] setting | | | | |
| Mean (SD) | 19.68 (36.2) | 19.35 (36.2) | 20.84 (36.9) | 0.609 |

© 2023 Richard L et al. *JAMA Network Open.*

| Participant demographics at baseline | | Total (n=736) | No evidence of past infection (n=512) | Evidence of past infection (n=224) | P-values |
|---|---|---|---|---|---|
| | Median (IQR) | 0.0 (0, 15) | 0.0 (0, 9) | 0.0 (0, 25) | 0.394 |
| % in Low Exposure[3] setting | | | | | |
| | Mean (SD) | 51.25 (46.4) | 52.14 (46.4) | 48.82 (46.5) | 0.373 |
| | Median (IQR) | 54.5 (0, 100) | 59.4 (0, 100) | 36.9 (0, 100) | 0.202 |
| Average number of people who shared living space | | | | | |
| | Mean (SD) | 5.38 (17.0) | 5.02 (16.2) | 6.20 (18.8) | 0.387 |
| | Median (IQR) | 0.5 (0, 2) | 0.3 (0, 2) | 0.9 (0, 3) | 0.279 |

SD=Standard deviation; IQR=Interquartile range; PHG=Public Health Guideline
[1] 'High exposure' includes time residing in a congregate homeless shelter, recovery centre, nursing home, jail or immigration detention centre
[2] 'Moderate exposure' includes time residing in a physical distancing hotel, non-congregate shelter, transitional housing, rooming house, encampment, on the street, rehab, hospital or 'other' settings.
[3] 'Low exposure' includes time residing in own home, supportive housing, private hotel/motel, or staying with friends and family

© 2023 Richard L et al. *JAMA Network Open.*

### 3B – Baseline characteristics of participants without history of infection at baseline, by follow-up status (>=1 follow-up; lost to follow-up)

| Participant demographics at baseline | | Total (n=512) | >1 Follow-up (n=415) | Lost to follow-up (n=97) | P-values |
|---|---|---|---|---|---|
| Age, mean (SD) | | 45.95 (14.4) | 46.61 (14.5) | 43.01 (14.0) | 0.027 |
| Age category, N (%) | | | | | |
| | 16-29 years old | 68 (13.28%) | 49 (11.81%) | 19 (19.59%) | 0.082 |
| | 30-49 years old | 230 (44.92%) | 183 (44.10%) | 47 (48.45%) | |
| | 50-69 years old | 185 (36.13%) | 158 (38.07%) | 27 (27.84%) | |
| | 70+ years old | 29 (5.66%) | 25 (6.02%) | 4 (4.12%) | |
| Self-reported gender, N (%) | | | | | |
| | Male | 334 (65.23%) | 272 (65.54%) | 62 (63.92%) | 0.182 |
| | Female | 163 (31.84%) | 134 (32.29%) | 29 (29.90%) | |
| | Other | 13 (2.54%) | 8 (1.93%) | 5 (5.15%) | |
| | Refused/Don't know | 2 (0.39%) | 1 (0.24%) | 1 (1.03%) | |
| Citizenship status, N (%) | | | | | |
| | Citizen | 398 (77.73%) | 319 (76.87%) | 46 (47.42%) | 0.436 |
| | Landed immigrant | 66 (12.89%) | 58 (13.98%) | 23 (23.71%) | |
| | Refugee claimant | 33 (6.45%) | 27 (6.51%) | 3 (3.09%) | |
| | Temporary/Other | 11 (2.15%) | 8 (1.93%) | 20 (20.62%) | |
| | Refused/Don't know | 4 (0.78%) | 3 (0.72%) | 1 (1.03%) | |
| Immigration history, N (%) | | | | | |
| | > 10 years ago | 139 (27.15%) | 113 (27.23%) | 26 (26.80%) | 0.946 |
| | <= 10 years ago | 68 (13.28%) | 56 (13.49%) | 12 (12.37%) | |
| | N/A (Born in Canada) | 305 (59.57%) | 246 (59.42%) | 59 (60.82%) | |
| Top education completed, N (%) | | | | | |
| | Less than high school | 149 (29.10%) | 121 (29.16%) | 28 (28.87%) | 0.384 |
| | High school | 183 (35.74%) | 144 (34.70%) | 39 (40.21%) | |
| | Post-secondary | 173 (33.79%) | 146 (35.18%) | 27 (27.84%) | |
| | Refused/Don't know | 7 (1.37%) | 4 (0.97%) | 3 (3.09%) | |
| One or more COVID-19 vaccine, N (%) | | | | | |

© 2023 Richard L et al. *JAMA Network Open.*

| Participant demographics at baseline | Total (n=512) | >1 Follow-up (n=415) | Lost to follow-up (n=97) | P-values |
|---|---|---|---|---|
| Yes | 420 (82.03%) | 343 (82.65%) | 77 (79.38%) | |
| No | 85 (16.60%) | 68 (16.39%) | 17 (17.53%) | 0.719 |
| Missing serology | 7 (1.37%) | 4 (0.97%) | 3 (3.09%) | |
| **Health behaviors during latest reported interval** | | | | |
| Paid or volunteer work, N (%) | 154 (30.08%) | 126 (30.36%) | 28 (28.87%) | 0.772 |
| Alcohol consumption | 332 (64.84%) | 264 (63.61%) | 68 (70.10%) | 0.157 |
| Alcohol consumption frequency | | | | |
| Never | 176 (34.38%) | 149 (35.90%) | 27 (27.84%) | |
| Monthly or less | 144 (28.13%) | 120 (28.92%) | 24 (24.74%) | |
| 2-4 times a month | 65 (12.70%) | 51 (12.29%) | 14 (14.43%) | 0.280 |
| 2-3 times a week | 59 (11.52%) | 46 (11.08%) | 13 (13.40%) | |
| 4+ times a week | 64 (12.50%) | 47 (11.33%) | 17 (17.53%) | |
| Refused/Don't know | 4 (0.78%) | 2 (0.48%) | 2 (2.06%) | |
| Tobacco consumption | 359 (70.12%) | 287 (69.16%) | 72 (74.23%) | 0.342 |
| Tobacco consumption frequency | | | | |
| Never | 152 (29.69%) | 127 (30.60%) | 25 (25.77%) | |
| Less than daily | 300 (58.59%) | 241 (58.07%) | 59 (60.82%) | 0.581 |
| Daily | 59 (11.52%) | 46 (11.08%) | 13 (13.40%) | |
| Refused/Don't know | 1 (0.20%) | 1 (0.24%) | 0 (0%) | |
| PHG #1: wears face mask in public | | | | |
| Good (Often or Always) | 440 (85.94%) | 365 (87.95%) | 75 (77.32%) | |
| Poor (Never, Rarely or Occasionally) | 68 (13.28%) | 47 (11.33%) | 21 (21.65%) | 0.024 |
| Refused/Don't know | 4 (0.78%) | 3 (0.72%) | 1 (1.03%) | |
| PHG #2: distances in public places | | | | |
| Good (Often or Always) | 435 (84.96%) | 362 (87.23%) | 73 (75.26%) | |
| Poor (Never, Rarely or Occasionally) | 70 (13.67%) | 50 (12.05%) | 20 (20.62%) | 0.002 |
| Refused/Don't know | 7 (1.37%) | 3 (0.72%) | 4 (4.12%) | |
| PHG #3: avoids crowded places or gatherings | | | | |
| Good (Often or Always) | 407 (79.49%) | 341 (82.17%) | 66 (68.04%) | 0.008 |

© 2023 Richard L et al. *JAMA Network Open.*

| Participant demographics at baseline | Total (n=512) | >1 Follow-up (n=415) | Lost to follow-up (n=97) | P-values |
|---|---|---|---|---|
| Poor (Never, Rarely or Occasionally) | 94 (18.36%) | 66 (15.90%) | 28 (28.87%) | |
| Refused/Don’t know | 11 (2.15%) | 8 (1.93%) | 3 (3.09%) | |
| PHG #4: washes hands with soap/sanitizer several times per day | | | | |
| Good (Often or Always) | 454 (88.67%) | 378 (91.08%) | 76 (78.35%) | |
| Poor (Never, Rarely or Occasionally) | 54 (10.55%) | 34 (8.19%) | 20 (20.62%) | 0.001 |
| Refused/Don’t know | 4 (0.78%) | 3 (0.72%) | 1 (1.03%) | |
| **Housing History in latest reported interval** | | | | |
| % in congregate shelter | | | | |
| Mean (SD) | 22.65 (37.6) | 23.42 (38.6) | 19.39 (33.3) | 0.344 |
| Median (IQR) | 0.0 (0, 30) | 0.0 (0, 32) | 0.0 (0, 20) | 0.496 |
| % in non-congregate shelter | | | | |
| Mean (SD) | 13.55 (31.5) | 13.94 (32.3) | 11.89 (27.9) | 0.565 |
| Median (IQR) | 0.0 (0, 0) | 0.0 (0, 0) | 0.0 (0, 3) | 0.384 |
| % in Physical Distancing Hotel | | | | |
| Mean (SD) | 38.54 (45.6) | 41.63 (46.3) | 25.31 (40.1) | 0.001 |
| Median (IQR) | 2.9 (0, 100) | 7.8 (0, 100) | 0.0 (0, 36) | 0.007 |
| % in own place | | | | |
| Mean (SD) | 6.84 (22.9) | 6.15 (21.6) | 9.80 (27.8) | 0.159 |
| Median (IQR) | 0.0 (0, 0) | 0.0 (0, 0) | 0.0 (0, 0) | 0.283 |
| % in High Exposure[1] setting | | | | |
| Mean (SD) | 24.01 (38.4) | 24.00 (39.0) | 24.07 (36.3) | 0.988 |
| Median (IQR) | 0.0 (0, 35) | 0.0 (0, 38) | 0.0 (0, 33) | 0.204 |
| % in Moderate Exposure[2] setting | | | | |
| Mean (SD) | 20.49 (36.1) | 19.16 (35.3) | 26.20 (39.1) | 0.084 |
| Median (IQR) | 0.0 (0, 25) | 0.0 (0, 18) | 0.0 (0, 58) | 0.032 |
| % in Low Exposure[3] setting | | | | |
| Mean (SD) | 53.31 (45.8) | 54.96 (45.8) | 46.22 (45.0) | 0.090 |
| Median (IQR) | 68.5 (0, 100) | 75.0 (0, 100) | 35.7 (0, 100) | 0.050 |
| Average number of people who shared living space | | | | |

© 2023 Richard L et al. *JAMA Network Open.*

| Participant demographics at baseline | Total (n=512) | >1 Follow-up (n=415) | Lost to follow-up (n=97) | P-values |
|---|---|---|---|---|
| Mean (SD) | 4.01 (11.7) | 3.69 (10.1) | 5.43 (16.8) | 0.188 |
| Median (IQR) | 0.4 (0, 2) | 0.2 (0, 2) | 0.8 (0, 2) | 0.181 |

SD=Standard deviation; IQR=Interquartile range; PHG=Public Health Guideline
[1] 'High exposure' includes time residing in a congregate homeless shelter, recovery centre, nursing home, jail or immigration detention centre
[2] 'Moderate exposure' includes time residing in a physical distancing hotel, non-congregate shelter, transitional housing, rooming house, encampment, on the street, rehab, hospital or 'other' settings.
[3] 'Low exposure' includes time residing in own home, supportive housing, private hotel/motel, or staying with friends and family

© 2023 Richard L et al. *JAMA Network Open.*

### 3C – Baseline characteristics of participants by inclusion in the main analysis (included; lost to follow-up/infected at baseline)

| Participant demographics at baseline | | Total (n=736) | Included (n=415) | Excluded (n=321) | P-values |
|---|---|---|---|---|---|
| Age, mean (SD) | | 46.08 (14.6) | 46.61 (14.5) | 45.40 (14.8) | 0.263 |
| Age category, N (%) | | | | | |
| | 16-29 years old | 100 (13.59%) | 49 (11.81%) | 51 (15.89%) | 0.275 |
| | 30-49 years old | 326 (44.29%) | 183 (44.10%) | 143 (44.55%) | |
| | 50-69 years old | 263 (35.73%) | 158 (38.07%) | 105 (32.71%) | |
| | 70+ years old | 47 (6.39%) | 25 (6.02%) | 22 (6.85%) | |
| Self-reported gender, N (%) | | | | | |
| | Male | 486 (66.03%) | 272 (65.54%) | 214 (66.67%) | 0.643 |
| | Female | 231 (31.39%) | 134 (32.29%) | 97 (30.22%) | |
| | Other | 17 (2.31%) | 8 (1.93%) | 9 (2.80%) | |
| | Refused/Don't know | 2 (0.27%) | 1 (0.24%) | 1 (0.31%) | |
| Citizenship status, N (%) | | | | | |
| | Citizen | 564 (76.63%) | 319 (76.87%) | 245 (76.32%) | 0.047 |
| | Landed immigrant | 90 (12.23%) | 58 (13.98%) | 32 (9.97%) | |
| | Refugee claimant | 55 (7.47%) | 27 (6.51%) | 28 (8.72%) | |
| | Temporary/Other | 20 (2.72%) | 8 (1.93%) | 12 (3.74%) | |
| | Refused/Don't know | 7 (0.95%) | 3 (0.72%) | 4 (2.24%) | |
| Immigration history, N (%) | | | | | |
| | > 10 years ago | 187 (25.41%) | 113 (27.23%) | 74 (23.05%) | 0.331 |
| | <= 10 years ago | 108 (14.67%) | 56 (13.49%) | 52 (16.20%) | |
| | N/A (Born in Canada) | 441 (59.92%) | 246 (59.28%) | 195 (60.75%) | |
| Top education completed, N (%) | | | | | |
| | Less than high school | 208 (28.26%) | 121 (29.16%) | 87 (27.10%) | 0.394 |
| | High school | 255 (34.65%) | 144 (34.70%) | 111 (34.58%) | |
| | Post-secondary | 264 (35.87%) | 146 (35.18%) | 118 (36.76%) | |
| | Refused/Don't know | 9 (1.23%) | 4 (0.97%) | 5 (1.55%) | |
| One or more COVID-19 vaccine, N (%) | | | | | |
| | Yes | 585 (79.48%) | 343 (82.65%) | 242 (75.39%) | 0.030 |

© 2023 Richard L et al. *JAMA Network Open.*

| Participant demographics at baseline | Total (n=736) | Included (n=415) | Excluded (n=321) | P-values |
|---|---|---|---|---|
| No | 143 (19.43%) | 68 (16.39%) | 75 (23.36%) | |
| Missing serology | 8 (1.09%) | 4 (0.97%) | 4 (1.24%) | |
| **Health behaviors during latest reported interval** | | | | |
| Paid or volunteer work, N (%) | 215 (29.21%) | 120 (28.92%) | 96 (29.91%) | 0.770 |
| Alcohol consumption | 449 (61.01%) | 237 (57.11%) | 211 (65.73%) | 0.027 |
| Alcohol consumption frequency | | | | |
| Never | 282 (38.32%) | 177 (42.65%) | 106 (33.02%) | 0.119 |
| Monthly or less | 174 (23.64%) | 94 (22.65%) | 79 (24.61%) | |
| 2-4 times a month | 100 (13.59%) | 56 (13.49%) | 44 (13.71%) | |
| 2-3 times a week | 79 (10.73%) | 41 (9.88%) | 38 (11.84%) | |
| 4+ times a week | 96 (13.04%) | 46 (11.08%) | 50 (15.58%) | |
| Refused/Don't know | 5 (0.68%) | 1 (0.24%) | 4 (1.24%) | |
| Tobacco consumption | 504 (68.48%) | 283 (68.19%) | 221 (68.85%) | 0.666 |
| Tobacco consumption frequency | | | | |
| Never | 230 (31.25%) | 131 (31.57%) | 99 (30.84%) | 0.845 |
| Less than daily | 435 (59.10%) | 244 (58.80%) | 191 (59.50%) | |
| Daily | 69 (9.38%) | 39 (9.40%) | 30 (9.35%) | |
| Refused/Don't know | 2 (0.28%) | 1 (0.24%) | 1 (0.31%) | |
| PHG #1: wears face mask in public | | | | |
| Good (Often or Always) | 651 (88.45%) | 380 (91.57%) | 271 (84.42%) | 0.005 |
| Poor (Never, Rarely or Occasionally) | 83 (11.28%) | 35 (8.43%) | 48 (14.95%) | |
| Refused/Don't know | 2 (0.27%) | 0 (0%) | 2 (0.62%) | |
| PHG #2: distances in public places | | | | |
| Good (Often or Always) | 625 (84.92%) | 364 (87.71%) | 260 (81.00%) | 0.014 |
| Poor (Never, Rarely or Occasionally) | 102 (13.86%) | 49 (11.81%) | 54 (16.82%) | |
| Refused/Don't know | 9 (1.22%) | 2 (0.48%) | 7 (2.18%) | |
| PHG #3: avoids crowded places or gatherings | | | | |
| Good (Often or Always) | 583 (79.21%) | 340 (81.93%) | 242 (75.39%) | 0.094 |
| Poor (Never, Rarely or Occasionally) | 140 (19.02%) | 69 (16.63%) | 72 (22.43%) | |

© 2023 Richard L et al. *JAMA Network Open.*

| Participant demographics at baseline | Total (n=736) | Included (n=415) | Excluded (n=321) | P-values |
|---|---|---|---|---|
| Refused/Don't know | 13 (1.77%) | 6 (1.45%) | 7 (2.18%) | |
| PHG #4: washes hands with soap/sanitizer several times per day | | | | |
| Good (Often or Always) | 663 (90.08%) | 385 (92.77%) | 278 (86.60%) | |
| Poor (Never, Rarely or Occasionally) | 70 (9.51%) | 29 (6.99%) | 41 (12.77%) | 0.020 |
| Refused/Don't know | 3 (0.41%) | 1 (0.24%) | 2 (0.62%) | |
| **Housing History in latest reported interval** | | | | |
| % in congregate shelter | | | | |
| Mean (SD) | 26.53 (40.6) | 27.88 (41.4) | 24.78 (39.5) | 0.305 |
| Median (IQR) | 0.0 (0, 57) | 0.0 (0, 70) | 0.0 (0, 36) | 0.686 |
| % in non-congregate shelter | | | | |
| Mean (SD) | 13.46 (31.5) | 13.94 (32.6) | 13.11 (30.5) | 0.724 |
| Median (IQR) | 0.0 (0, 0) | 0.0 (0, 0) | 0.0 (0, 0) | 0.220 |
| % in Physical Distancing Hotel | | | | |
| Mean (SD) | 40.83 (46.3) | 45.57 (47.0) | 34.71 (44.7) | 0.002 |
| Median (IQR) | 5.9 (0, 100) | 22.7 (0, 100) | 0.0 (0, 100) | 0.009 |
| % in own place | | | | |
| Mean (SD) | 5.73 (20.2) | 5.58 (18.7) | 5.64 (21.6) | 0.970 |
| Median (IQR) | 0.0 (0, 0) | 0.0 (0, 0) | 0.0 (0, 0) | 0.098 |
| % in High Exposure[1] setting | | | | |
| Mean (SD) | 27.75 (41.2) | 28.15 (41.6) | 27.23 (40.7) | 0.765 |
| Median (IQR) | 0.0 (0, 69) | 0.0 (0, 70) | 0.0 (0, 63) | 0.363 |
| % in Moderate Exposure[2] setting | | | | |
| Mean (SD) | 19.68 (36.2) | 17.77 (35.3) | 22.43 (37.6) | 0.085 |
| Median (IQR) | 0.0 (0, 15) | 0.0 (0, 4) | 0.0 (0, 32) | 0.011 |
| % in Low Exposure[3] setting | | | | |
| Mean (SD) | 51.25 (46.4) | 53.52 (46.6) | 48.04 (46.0) | 0.111 |
| Median (IQR) | 54.5 (0, 100) | 64.2 (0, 100) | 36.9 (0, 100) | 0.023 |
| Average number of people who shared living space | | | | |
| Mean (SD) | 5.38 (17.0) | 4.92 (16.1) | 5.97 (18.2) | 0.411 |

© 2023 Richard L et al. *JAMA Network Open.*

| Participant demographics at baseline | Total (n=736) | Included (n=415) | Excluded (n=321) | P-values |
|---|---|---|---|---|
| Median (IQR) | 0.5 (0, 2) | 0.1 (0, 2) | 0.9 (0, 3) | 0.077 |

SD=Standard deviation; IQR=Interquartile range; PHG=Public Health Guideline

[1] 'High exposure' includes time residing in a congregate homeless shelter, recovery centre, nursing home, jail or immigration detention centre

[2] 'Moderate exposure' includes time residing in a physical distancing hotel, non-congregate shelter, transitional housing, rooming house, encampment, on the street, rehab, hospital or 'other' settings.

[3] 'Low exposure' includes time residing in own home, supportive housing, private hotel/motel, or staying with friends and family

© 2023 Richard L et al. *JAMA Network Open.*

**eAppendix 4. Characteristics of Participants Without History of Infection at Baseline, by Incident Infection Status by 6 Months (415 Participants; 716 Intervals)**

| Participant characteristics reported at baseline | | Total (n=415) | No incident infection (n=291) | Incident infection (n=124) |
|---|---|---|---|---|
| Age, mean (SD) | | 46.64 (14.5) | 46.26 (13.9) | 47.52 (15.7) |
| Age category, N (%) | | | | |
| | 16-29 years old | 49 (11.81%) | 37 (12.71%) | 12 (9.68%) |
| | 30-49 years old | 183 (44.10%) | 124 (42.61%) | 59 (47.58%) |
| | 50-69 years old | 158 (38.07%) | 115 (39.52%) | 43 (34.68%) |
| | 70+ years old | 25 (6.02%) | 15 (5.15%) | 10 (8.06%) |
| Self-reported gender, N (%) | | | | |
| | Male | 272 (65.54%) | 185 (63.57%) | 87 (70.16%) |
| | Female | 134 (32.29%) | 99 (34.02%) | 35 (28.23%) |
| | Other | 8 (1.93%) | 6 (2.06%) | 2 (1.61%) |
| | Refused/Don’t know | 1 (0.20%) | 1 (0.40%) | 0 (0.00%) |
| Citizenship status, N (%) | | | | |
| | Citizen | 319 (76.87%) | 228 (78.35%) | 91 (73.39%) |
| | Landed immigrant | 58 (13.98%) | 38 (13.06%) | 20 (16.13%) |
| | Refugee claimant | 27 (6.51%) | 19 (6.53%) | 8 (6.45%) |
| | Temporary/Other | 8 (1.93%) | 5 (1.72%) | 3 (2.42%) |
| | Refused/Don’t know | 3 (0.70%) | 1 (0.40%) | 2 (1.60%) |
| Immigration history, N (%) | | | | |
| | > 10 years ago | 113 (27.23%) | 74 (25.43%) | 39 (31.45%) |
| | <= 10 years ago | 56 (13.49%) | 31 (10.65%) | 25 (20.16%) |
| | N/A (Born in Canada) | 246 (59.28%) | 186 (63.92%) | 60 (48.39%) |
| Top education completed, N (%) | | | | |
| | Less than high school | 121 (29.16%) | 82 (28.18%) | 39 (31.45%) |
| | High school | 144 (34.70%) | 99 (34.02%) | 45 (36.29%) |
| | Post-secondary | 146 (35.18%) | 108 (37.11%) | 38 (30.65%) |
| | Refused/Don’t know | 4 (1.00%) | 2 (0.70%) | 2 (1.60%) |
| Comorbidities, N (%) | | | | |
| | Hypertension | 67 (16.14%) | 45 (15.46%) | 22 (17.74%) |

© 2023 Richard L et al. *JAMA Network Open.*

| Participant characteristics reported at baseline | Total (n=415) | No incident infection (n=291) | Incident infection (n=124) |
|---|---|---|---|
| Diabetes | 51 (12.29%) | 38 (13.06%) | 13 (10.48%) |
| Asthma | 88 (21.20%) | 65 (22.34%) | 23 (18.55%) |
| Lung disease (COPD, emphysema, or chronic bronchitis) | 36 (8.67%) | 28 (9.62%) | 8 (6.45%) |
| Heart disease (heart attack, heart failure, or coronary artery disease) | 20 (4.82%) | 14 (4.81%) | 6 (4.84%) |
| History of stroke | 17 (4.10%) | 11 (3.78%) | 6 (4.84%) |
| Chronic kidney disease | 9 (2.17%) | 6 (2.06%) | 3 (2.42%) |
| Liver disease | 23 (5.54%) | 18 (6.19%) | 5 (4.03%) |
| Chronic neurological disorder | 30 (7.23%) | 22 (7.56%) | 8 (6.45%) |
| Cancer | 24 (5.78%) | 13 (4.47%) | 11 (8.87%) |
| HIV/AIDS | 7 (1.69%) | 6 (2.06%) | 1 (0.81%) |
| Immune Suppressed (other than HIV/AIDS) | 13 (3.13%) | 9 (3.09%) | 4 (3.23%) |
| COVID-19 vaccines received by the baseline interview, N (%) | | | |
| None | 78 (18.80%) | 59 (20.27%) | 19 (15.32%) |
| Incomplete primary series | 68 (16.39%) | 45 (15.46%) | 23 (18.55%) |
| Complete primary series (2 dose or 1 dose of Johnson & Johnson) | 265 (63.86%) | 184 (63.23%) | 81 (65.32%) |
| Complete primary series and booster | 4 (0.96%) | 3 (1.03%) | 1 (0.81%) |
| **Health behaviors during latest reported interval** | **Total (n=721)** | **No seroconversion (n=597)** | **Seroconversion (n=124)** |
| Paid or volunteer work, N (%) | 205 (28.43%) | 167 (27.97%) | 38 (30.65%) |
| Alcohol consumption, N (%) | 408 (56.59%) | 326 (54.61%) | 82 (66.13%) |
| Alcohol consumption frequency, N (%) | | | |
| Never | 311 (43.13%) | 270 (45.23%) | 41 (33.06%) |
| Monthly or less | 168 (23.30%) | 133 (22.28%) | 35 (28.23%) |
| 2-4 times a month | 95 (13.18%) | 74 (12.40%) | 21 (16.94%) |
| 2-3 times a week | 70 (9.71%) | 53 (8.88%) | 17 (13.71%) |
| 4+ times a week | 75 (10.40%) | 66 (11.06%) | 9 (7.26%) |
| Refused/Don’t know | 2 (0.30%) | 1 (0.20%) | 1 (0.80%) |
| Tobacco consumption, N (%) | 474 (65.74%) | 395 (66.16%) | 79 (63.71%) |
| Tobacco consumption frequency, N (%) | | | |
| Never | 245 (33.98%) | 201 (33.67%) | 44 (35.48%) |

© 2023 Richard L et al. *JAMA Network Open.*

| Participant characteristics reported at baseline | Total (n=415) | No incident infection (n=291) | Incident infection (n=124) |
|---|---|---|---|
| Less than daily | 60 (8.32%) | 45 (7.54%) | 15 (12.10%) |
| Daily | 414 (57.42%) | 350 (58.63%) | 64 (51.61%) |
| Refused/Don't know | 2 (0.3%) | 1 (0.2%) | 1 (0.8%) |
| Consumption of illegal or prescription medication for non-medical reasons, N (%) | 158 (21.91%) | 132 (22.11%) | 26 (20.97%) |
| PHG #1: wears face mask in public | | | |
| Good (Often or Always) | 666 (92.37%) | 554 (92.80%) | 112 (90.32%) |
| Poor (Never, Rarely or Occasionally) | 55 (7.63%) | 43 (7.20%) | 12 (9.68%) |
| PHG #2: distances in public places | | | |
| Good (Often or Always) | 646 (89.60%) | 537 (89.95%) | 109 (87.90%) |
| Poor (Never, Rarely or Occasionally) | 72 (9.99%) | 59 (9.88%) | 13 (10.48%) |
| Refused/Don't know | 3 (0.40%) | 1 (0.20%) | 2 (1.60%) |
| PHG #3: avoids crowded places or gatherings | | | |
| Good (Often or Always) | 594 (82.39%) | 492 (82.41%) | 102 (82.26%) |
| Poor (Never, Rarely or Occasionally) | 118 (16.37%) | 97 (16.25%) | 21 (16.94%) |
| Refused/Don't know | 9 (1.30%) | 8 (1.40%) | 1 (0.80%) |
| PHG #4: washes hands with soap/sanitizer several times per day | | | |
| Good (Often or Always) | 667 (92.51%) | 551 (92.29%) | 116 (93.55%) |
| Poor (Never, Rarely or Occasionally) | 52 (7.21%) | 44 (7.37%) | 8 (6.45%) |
| Refused/Don't know | 2 (0.28%) | 2 (0.3%) | 0 (0.0%) |
| **Housing History in latest reported interval** | **Total (n=721)** | **No seroconversion (n=597)** | **Seroconversion (n=124)** |
| % in congregate shelter | | | |
| Mean (SD) | 25.52 (40.8) | 24.77 (40.5) | 29.15 (42.1) |
| Median (IQR) | 0.0 (0, 55) | 0.0 (0, 45) | 0.0 (0, 70) |
| % in non-congregate shelter | | | |
| Mean (SD) | 13.32 (32.3) | 14.48 (33.6) | 7.71 (24.2) |
| Median (IQR) | 0.0 (0, 0) | 0.0 (0, 0) | 0.0 (0, 0) |
| % in Physical Distancing Hotel | | | |
| Mean (SD) | 45.86 (47.3) | 45.58 (47.3) | 47.22 (47.3) |

© 2023 Richard L et al. *JAMA Network Open.*

| Participant characteristics reported at baseline | | Total (n=415) | No incident infection (n=291) | Incident infection (n=124) |
|---|---|---|---|---|
| | Median (IQR) | 22.7 (0, 100) | 18.8 (0, 100) | 43.1 (0, 100) |
| % in own place | | | | |
| | Mean (SD) | 8.68 (25.5) | 8.92 (26.0) | 7.50 (22.9) |
| | Median (IQR) | 0.0 (0, 0) | 0.0 (0, 0) | 0.0 (0, 0) |
| % in High Exposure[1] setting | | | | |
| | Mean (SD) | 25.74 (40.9) | 25.04 (40.6) | 29.15 (42.1) |
| | Median (IQR) | 0.0 (0, 57) | 0.0 (0, 47) | 0.0 (0, 70) |
| % in Moderate Exposure[2] setting | | | | |
| | Mean (SD) | 16.06 (34.3) | 17.02 (35.3) | 11.45 (28.9) |
| | Median (IQR) | 0.0 (0, 0) | 0.0 (0, 0) | 0.0 (0, 0) |
| % in Low Exposure[3] setting | | | | |
| | Mean (SD) | 57.31 (46.8) | 57.22 (46.9) | 57.74 (46.5) |
| | Median (IQR) | 100.0 (0, 100) | 100.0 (0, 100) | 100.0 (0, 100) |
| Number of moves during period | | | | |
| | Mean (SD) | 3.56 (4.5) | 3.46 (4.4) | 4.03 (5.0) |
| | Median (IQR) | 2.0 (1, 4) | 2.0 (1, 4) | 3.0 (1, 5) |
| Average number of people who shared living space | | | | |
| | Mean (SD) | 4.34 (14.5) | 4.05 (14.1) | 5.72 (16.4) |
| | Median (IQR) | 0.0 (0, 2) | 0.0 (0, 2) | 1.0 (0, 4) |
| Interview after onset of Omicron as dominant variant, N (%) | | | | |
| | No | 410 (56.87%) | 390 (65.33%) | 20 (16.13%) |
| | Yes | 311 (43.13%) | 207 (34.67%) | 104 (83.87%) |

SD=Standard deviation; IQR=Interquartile range; PHG=Public Health Guideline; COPD= chronic obstructive pulmonary disease

[1] 'High exposure' includes time residing in a congregate homeless shelter, recovery centre, nursing home, jail or immigration detention centre

[2] 'Moderate exposure' includes time residing in a physical distancing hotel, non-congregate shelter, transitional housing, rooming house, encampment, on the street, rehab, hospital or 'other' settings.

[3] 'Low exposure' includes time residing in own home, supportive housing, private hotel/motel, or staying with friends and family

© 2023 Richard L et al. *JAMA Network Open.*

**eReferences.**


1. Richard L, Nisenbaum R, Liu M, et al. *Ku-gaa-gii pimitiziwin*, the COVID-19 Cohort Study of People Experiencing Homelessness in Toronto, Canada: A study protocol. *BMJ Open.* 2022;12. doi: 10.1136/bmjopen-2022-063234
2. Wyllie AL, Fournier J, Casanovas-Massana A, et al. Saliva or Nasopharyngeal Swab Specimens for Detection of SARS-CoV-2. *N Engl J Med.* 2020;383(13):1283-86. doi: 10.1056/NEJMc2016359
3. Colwill K, Galipeau Y, Stuible M, et al. A scalable serology solution for profiling humoral immune responses to COVID-19 infection and vaccination. *Clinical & Translational Immunology*. 2022;11(3). doi: 10.1002/cti2.1380
