# Supplementary material for: Incidence of SARS-CoV-2 Infection Among People Experiencing Homelessness in Toronto, Canada
Source: JAMA Netw Open. 2023 Mar 13;6(3):e232774. doi: 10.1001/jamanetworkopen.2023.2774 (PMC10011938; doi:10.1001/jamanetworkopen.2023.2774)
Supplement: Supplement 2. — Data Sharing Statement [file jamanetwopen-e232774-s002.pdf]

Data Sharing Statement

Data
Data available: No

Additional Information
Explanation for why data not available: The Ku-gaa-gii pimitizi-win study includes sensitive data pertaining to a marginalized group that includes Indigenous participants. A data dictionary detailing each variable used in this analysis and summary statistics for the variables used in this analysis are available in the Supplement. Requests to access additional summary or individual-level data can be directed to the data owners and possessors (for non-Indigenous participants: the study principal investigator Dr. Stephen Hwang; for Indigenous participants: Anishnawbe Health Toronto).
